# Non-invasive classification of non-small cell lung cancer: a comparison between random forest models utilising radiomic and semantic features

**DOI:** 10.1259/bjr.20190159

**Published:** 2019-06-03

**Authors:** Usman Bashir, Bhavin Kawa, Muhammad Siddique, Sze Mun Mak, Arjun Nair, Emma Mclean, Andrea Bille, Vicky Goh, Gary Cook

**Affiliations:** 1 Cancer Imaging Department, School of Biomedical Engineering and Imaging Sciences, King's College London, London, UK; 2 Department of Radiology, Maidstone Hospital, Hermitage Lane, Maidstone, UK; 3 Department of Radiology, Guy’s Hospital, Great Maze Pond, London, UK; 4 Department of Pathology, Guy’s Hospital and St Thomas’ NHS Foundation Trust, Westminster Bridge Rd, Lambeth, London, UK; 5 Department of Thoracic Surgery, Guy’s Hospital, Great Maze Pond, London, UK; 6 PET Imaging Centre and the Division of Imaging Sciences and Biomedical Engineering, King’s College London,, UK

## Abstract

**Objective:**

Non-invasive distinction between squamous cell carcinoma and adenocarcinoma subtypes of non-small-cell lung cancer (NSCLC) may be beneficial to patients unfit for invasive diagnostic procedures or when tissue is insufficient for diagnosis. The purpose of our study was to compare the performance of random forest algorithms utilizing CT radiomics and/or semantic features in classifying NSCLC.

**Methods:**

Two thoracic radiologists scored 11 semantic features on CT scans of 106 patients with NSCLC. A set of 115 radiomics features was extracted from the CT scans. Random forest models were developed from semantic (RM-sem), radiomics (RM-rad), and all features combined (RM-all). External validation of models was performed using an independent test data set (*n* = 100) of CT scans. Model performance was measured with out-of-bag error and area under curve (AUC), and compared using receiver-operating characteristics curve analysis on the test data set.

**Results:**

The median (interquartile-range) error rates of the models were: RF-sem 24.5 % (22.6 – 37.5 %), RF-rad 35.8 % (34.9 – 38.7 %), and RM-all 37.7 % (37.7 – 37.7). On training data, both RF-rad and RF-all gave perfect discrimination (AUC = 1), which was significantly higher than that achieved by RF-sem (AUC = 0.78; *p* < 0.0001). On test data, however, RM-sem model (AUC = 0.82) out-performed RM-rad and RM-all (AUC = 0.5 and AUC = 0.56; *p* < 0.0001), neither of which was significantly different from random guess ( *p* = 0.9 and 0.6 respectively).

**Conclusion:**

Non-invasive classification of NSCLC can be done accurately using random forest classification models based on well-known CT-derived descriptive features. However, radiomics-based classification models performed poorly in this scenario when tested on independent data and should be used with caution, due to their possible lack of generalizability to new data.

**Advances in knowledge:**

Our study describes novel CT-derived random forest models based on radiologist-interpretation of CT scans (semantic features) that can assist NSCLC classification when histopathology is equivocal or when histopathological sampling is not possible. It also shows that random forest models based on semantic features may be more useful than those built from computational radiomic features.

## Introduction

Non-small cell lung cancers (NSCLCs) comprise 85% of all primary lung malignancies.^1^ Of these, approximately 60% are adenocarcinomas (ADCA) and 35–40% are squamous cell carcinomas (SCCA), with large cell cancers accounting for less than 5%.^1^ Conventionally, ADCA and SCCA are differentiated by histopathological examination of haematoxylin & eosin-stained slides. ADCAs, depending upon the predominant pathologic subtype, may exhibit lepidic, glandular, papillary or micropapillary, or solid sheet-like architecture. SCCAs are characterized by the presence of keratinization, pearl formation, and intercellular bridges.^2^ Frequently, NSCLC is diagnosed on sputum cytology or clinical and radiological features, but adequate tissue is not available to perform histological subtyping and molecular analysis, requiring a multidisciplinary approach for decision-making.^2^ Although curative options for both NSCLC subtypes are similar—either surgical or with stereotactic body radiotherapy (SABR—the two subtypes differ in prognosis and choice of targeted agents.^3^ Hence, an accurate non-invasive test for NSCLC classification could serve as a valuable alternative for prognostication and choosing targeted agents in patients unsuitable for surgical resection.

Radiomics and machine learning (ML) are becoming increasingly popular in imaging research.^4^ Radiomics involves computational analysis of a greyscale image to derive features (*e.g.* mean, mode, kurtosis, and skewness) which are expected to quantify the tumour pathophysiology.^5^ ML is the task of using radiomics and other relevant variables (*e.g.* age, sex, and air bronchogram) in suitable computational algorithms (*e.g.* random forests or logistic regression) to infer clinically relevant information, *e.g.* tumour subtype. CT radiomics has been shown to be moderately to highly accurate in predicting NSCLC subtype, with reported performance of 68–90%.^6–8^ However, despite the potential of radiomics in changing imaging paradigms,^5^ widespread acceptance of radiomics is hindered by largely unmet challenges surrounding variable reproducibility, procedure standardization, and biologic explanation of used variables.^4,9,10^

Semantic features, *i.e*. features derived from subjective interpretation of CT images by a radiologist, have been shown to be related to tumour subtype and histopathology in numerous independent studies.^11–17^ Air-bronchogram and ground-glass opacification are more common in ADCA, whereas cavitation and spiculation are more common in SCCA.^16,17^ To our knowledge however, despite these well-known associations, semantic features have not been modelled in ML algorithms to predict tumour subtype and therefore help clinical decision making in a quantitative manner. Furthermore, no studies have compared or combined radiomic features with semantic features (*e.g.* air bronchogram and cavitation) in differentiating ADCA from SCCA.

We hypothesized that multivariate predictive models combining the strengths of semantic and radiomic features could yield potentially higher accuracy in NSCLC classification than either class of variables alone. Such non-invasive classification would benefit patients for whom an adequate histopathological subtyping cannot be obtained. Therefore, the objective of this study was to develop and compare NSCLC classification models based on semantic features, radiomic features, and a combination of both.

## methods and patients

### Patient population

**Figure 1.**
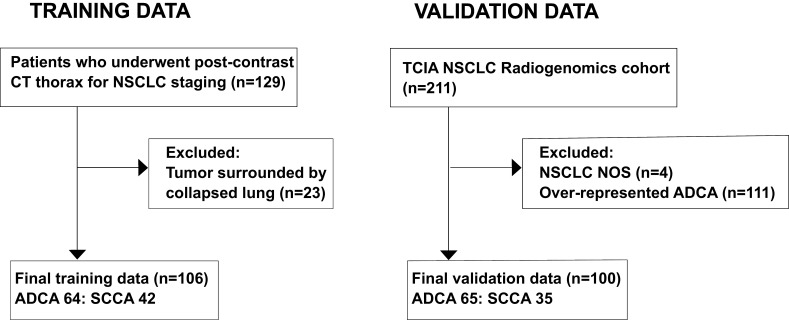
Patient inclusion workflow in our study for training and validation data sets. ADCA, adenocarcinoma; NOS, not otherwise specified; NSCLC, non-small-cell lung cancer; SCCA, squamous cell carcinoma; TCIA, The cancer imaging Archive.

The training data set comprised patients referred to a single institution as follows: we identified pre-treatment CT scans of pathologically proved NSCLC patients referred to our tertiary care centre from January 1, 2011 to December 31, 2015. Patients were excluded if it was not possible to accurately determine tumour boundaries on CT, *e.g.* due to adjacent atelectasis. The final data set comprised 106 studies (42 SCCA, 64 ADCA; Figure 1). The independent validation cohort (*n* = 100) comprised 65 ADCAs and 35 SCCAs downloaded from the Cancer Imaging archive, subsampled with respect to ADCAs to ensure balanced proportions.^18–20^ Local ethics committee waived informed written consent for this retrospective study of anonymised data.

### Imaging

Imaging of patients in the training data set was performed on one of three Philips scanners: MX8000, Brilliance iCT 256, or Brilliance 40 (Philips Medical Systems, Best, Netherlands). Patients were imaged in the supine position at full inspiration. Scanning parameters were as follows: detector collimation: 0.625–0.75; rotation time: 0.5–0.75 s; tube voltage: 120 kVp; tube current: 34–229 mAs. 100–150 ml iopromide 300 (300 mg I/mL Ultravist, Bayer Pharma, Berlin, Germany) was administered intravenously at a rate of 2–4 ml s^−1^ after a 30–70 s delay.

### Semantic features

Two thoracic radiologists (AN and MM, with 14 and 9 years’ experience, respectively), blinded to histopathological diagnosis, independently recorded nine nodule semantic feature (Table 1) and two background parenchymal features, *i.e.* emphysema (present or absent) and airway thickening (present or absent).^11,12,21–26^

**Table 1.** Nodule semantic features and their descriptions

**Semantic feature**	**Description**
**Air-bronchogram**	Presence of visible air-filled bronchi within the lesion. Measured as being present or absent.
**Ground-glass component**	Presence of hazy attenuation, higher than background, but not sufficiently high to obscure bronchial and vascular margins within the lesion.^21^
**Location**	Central or peripheral, based on whether the tumour was closer to the hilum than the nearest segmental bronchus or not.
**Margins**	Irregular, smooth, or lobulated. Lobulation was defined as the presence of at least three undulations with a height of more than 2 mm.^21^
**Pleural indentation**	Retraction of pleura near the tumour margin.^22^
**Satellite nodules**	Presence of smaller nodules in the immediate vicinity of the main lesion.
**Spiculation**	The presence of linear strands at least 2 mm thick extending from tumour margin into adjacent parenchyma.^21,23^
**Cavitation**	Presence of a round lucency inside the lesion, usually within the centre of the lesion and larger than pseudo cavitation; suggests necrosis.^21^
Pseudocavitation	Presence of bubble-like areas of low attenuation within the nodule.

Discrepant findings were resolved by consensus. Annotation of the validation data set was performed by a separate blinded reader, UB (10 years’ radiology experience), using the same descriptions.

### Radiomic features

Tumours were delineated by UB open-source software ITK-Snap (v. 3.4.0; Supplementary Material 1).^27^ From the segmented volumes-of-interest, 756 radiomic features were derived using an in-house feature extraction tool developed in MATLAB (Release 2016b, The MathWorks, Inc., Natick, MA). Highly correlated redundant features (showing pairwise correlation coefficient >0.8; *n* = 641) were removed to yield a final set of independent 115 radiomic features.

### Random forest model development and validation

In this study, we used random forests for machine learning. Random forests are known for their high performance and generalizability.^28^ Here we present a summary of random forest model development; technical details are provided in the supplemental data.

A random forest model is a group of a large number of decision trees, *e.g.* 2000. The name “random“ alludes to the fact that each split of an individual decision tree is developed from a random subset of input variables. Each member tree is also trained on a slightly different variation of the data-set by using bootstrap sampling, *i.e.* sampling with replacement, whereby several cases are sampled more than once and others omitted altogether (labelled “out-of-bag” [OOB] samples). Since the OOB samples have not been used in training the particular tree, they are used for internal validation, and the proportion of misclassified cases in the OOB sample serves as a performance metric: OOB error. After training of all 2000 decision trees is complete, a new case is classified by the entire ‘random forest’ by obtaining votes from member trees. A decision threshold is set, based on the preferred degree of sensitivity, to provide a final classification for each new case; for example, using a 50% probability threshold, a case may be classified as ADCA if >50% trees classify it as ADCA, or SCCA otherwise.

We developed three random forest classifiers using the training data set: One classifier comprising semantic variables only (RF-sem), one comprising radiomic features only (RF-rad), and one comprising both semantic and radiomic features (RF-all). Model validation was performed on the independent validation cohort.

### Statistical analysis

R v. 3.3.2 was used for statistical analysis.^29^ Continuous variables were reported as means and standard deviations. For descriptive analysis, differences between ADCAs and SCCAs were determined using Wilcoxon ranked sum test for continuous variables and using Fisher’s exact test for categorical variables. Inter-observer agreement between the two radiologists with regards to semantic variables was measured with Cohen’s κ test and summarised as estimated weighted κ scores alongwith their 95% CIs. A *p*-value cut-off of 0.05 was used to determine statistical significance.

The performance of random forest models was reported in terms of two metrics: The OOB error of random forest models was reported as the error rate of decision trees during internal validation. The second metric—area under curve (AUC)—served as the performance metric of fully trained models and was reported separately for training and validation data. We used two metrics instead of one to illustrate both the robustness of individual trees (OOB error) and that of the forest as a whole (AUC). Both metrics are related, and an ideal classifier should have both a low OOB error and a high AUC.

Since our random forests used large numbers of variables, we also measured the importance of individual variables in the training data set using the “mean decrease in accuracy“ (MDA) metric, *i.e.* decrease in classifier accuracy by removing the variable in question. The higher the MDA of a variable the more important the variable is. A variable with MDA of zero has no association with the outcome (tumour subtype) and there is no decrease in classifier accuracy if that variable is removed. Variables with low but non-zero MDA are still useful since random forests by design work well when individual variables are weakly related to the outcome, and mitigate their weak association by pooling them into a robust final classifier.^28^

## Results

The mean interval between pathologic diagnosis and CT chest imaging was 21 days (range 5–41 days). Patients were aged from 40.3 to 85.5 years (median: 71.4 years), with similar gender proportions (50 females: 56 males). There were no significant differences between patients with ADCA *vs* SCCA in terms of age (*p* = 0.6), smoking (*p* = 0.67), or gender (0.55) (Table 2).

**Table 2.** Clinical and demographic features of patients in training data set

**Clinical feature**	**ADCA**	**SCCA**
Age in years, mean (range, SD)	69 (40.2–84.75, 10.2)	70.8 (52.35–85.54,8.1)
Sex (M : F)	32 : 32	24 : 18
Smokers	65.6% (*n* = 42)	71.4%(*n* = 30)
T1a	10	7
T1b	12	6
T2a	27	15
T2b	3	5
T3	10	8
T4	2	1
N0	50	35
N1	3	3
N2	11	3
N3	0	1
M0	64	40
M1	0	2

ADCA, adenocarcinoma; SCCA, squamous cell carcinoma; SD = standard deviation.

Of the 13 tested semantic variables, three were significantly more common in ADCAs, *i.e*. air bronchogram (*p* < 0.0001), ground-glass component (*p* = 0.0006), and satellite nodules (*p* = 0.004). Cavitation was present in only 9 of the 106 total cases, of which 8 were SCCAs (*p* = 0.002). Table 3 describes the frequencies of semantic variables in both NSCLC subtypes.

**Table 3.** Frequencies of semantic features according to tumour type

	**Semantic feature**		**Tumour type**		**Fisher’s exact test**	**Interobserver agreement**
			ADCA (*n* = 64)	SCCA (*n* = 42)		Weighted-κ (95% CI)
**1**.	Air-bronchogram	Absent	31 (48.44%)	36 (85.71%)	<0.0001	0.34 (0.16 to 0.52)
		Present	33 (51.56%)	6 (14.29%)		
**2**.	Airway thickening	Absent	31 (48.44%)	15 (35.71%)	0.2	0.44 (0.25 to 0.63)
		Present	30 (46.88%)	20 (47.62%)		
**3**.	Emphysema	Absent	24 (37.5%)	10 (23.81%)	0.2	0.78 (0.69 to 0.86)
		Present	20 (31.25%)	16 (38.1%)		
**4**.	Ground-glass component	Absent	50 (78.13%)	42 (100%)	0.0006	0.74 (0.54 to 0.94)
		Present	14 (21.88%)	0 (0%)		
**5**.	Location	Central third	20 (31.25%)	10 (23.81%)	0.5	0.35 (0.16 to 0.55)
		Peripheral two-thirds	44 (68.75%)	32 (76.19%)		
**6**.	Margins	Irregular	35 (54.69%)	22 (52.38%)	0.9	0.2 (0.04 to 0.35)
		Lobulated	27 (42.19%)	18 (42.86%)		
		Smooth	2 (3.13%)	2 (4.76%)		
**7**.	Pleural indentation	Absent	18 (28.13%)	10 (23.81%)	0.65	0.44 (0.24 to 0.63)
		Present	46 (71.88%)	32 (76.19%)		
**8**.	Satellite nodules	Absent	50 (78.13%)	41 (97.62%)	0.004	0.74 (0.55 to 0.92)
		Present	14 (21.88%)	1 (2.38%)		
**9**.	Spiculation	Absent	38 (59.38%)	23 (54.76%)	0.69	0.27 (0.11 to 0.42)
		Present	26 (40.63%)	19 (45.24%)		
**10**.	Cavitation	Absent	63 (98.44%)	34 (80.95%)	0.002	0.78 (0.57 to 0.99)
		Present	1 (1.56%)	8 (19.05%)		
**11**.	Pseudocavitation	Absent	51 (79.69%)	39 (92.86%)	0.09	0.23 (0.01 to 0.45)
		Present	13 (20.31%)	3 (7.14%)		

IQR, interquartile range; SD, standard deviation.

### Comparison of random forest models

**Figure 2.**
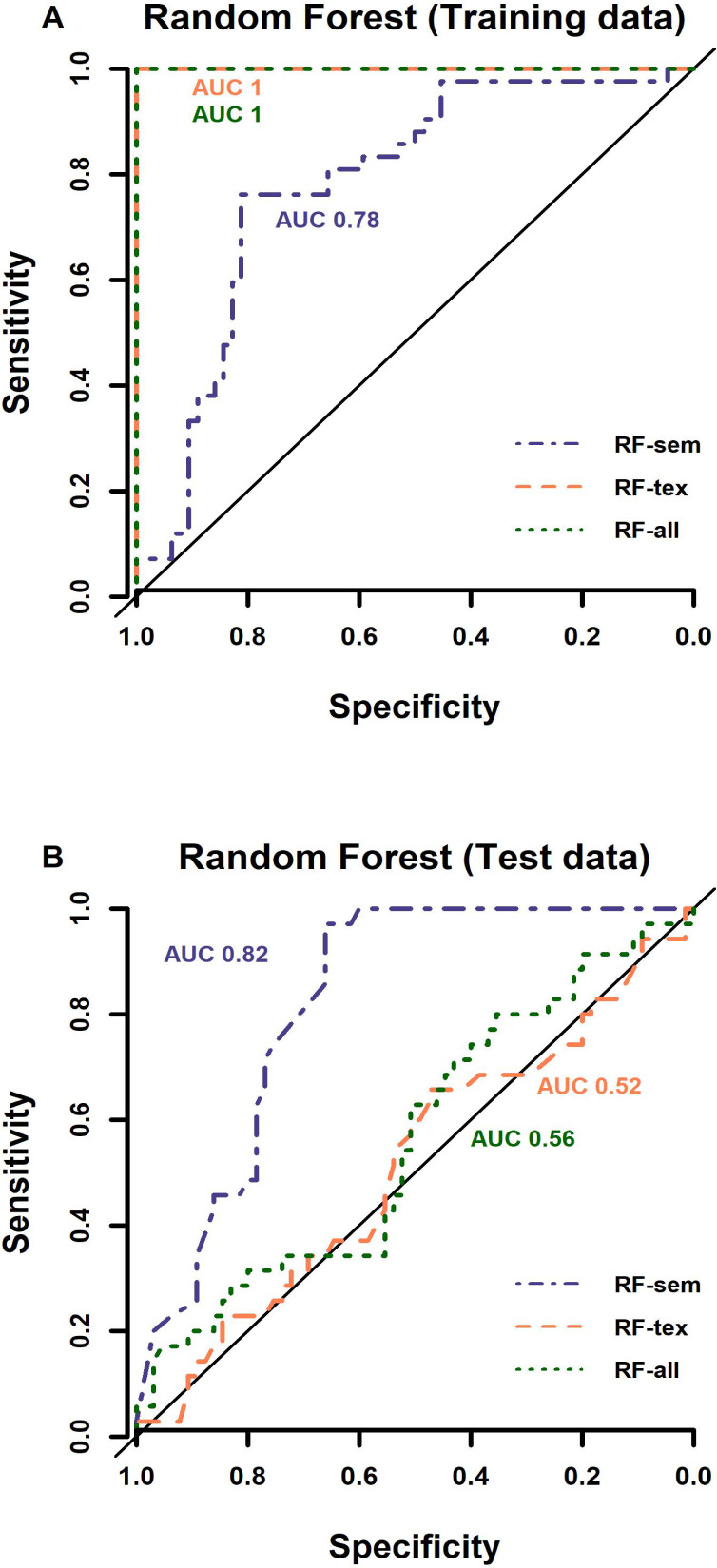
Performance curves of RF models on test data (A) and training data (B) show that RF models containing radiomic features (*i.e.* RF-rad and RF-all) yielded perfect discrimination (AUC 1) on training data (A), but very poor discrimination (AUC 0.52 and 0.56 respectively) on test data, similar to random guess (black line in A and B). RF-sem gave consistent good performance on training (B; AUC 0.78) as well as test data (B; AUC 0.82). AUC, area under the curve; RF, radiofrequency.

**Figure 3.**
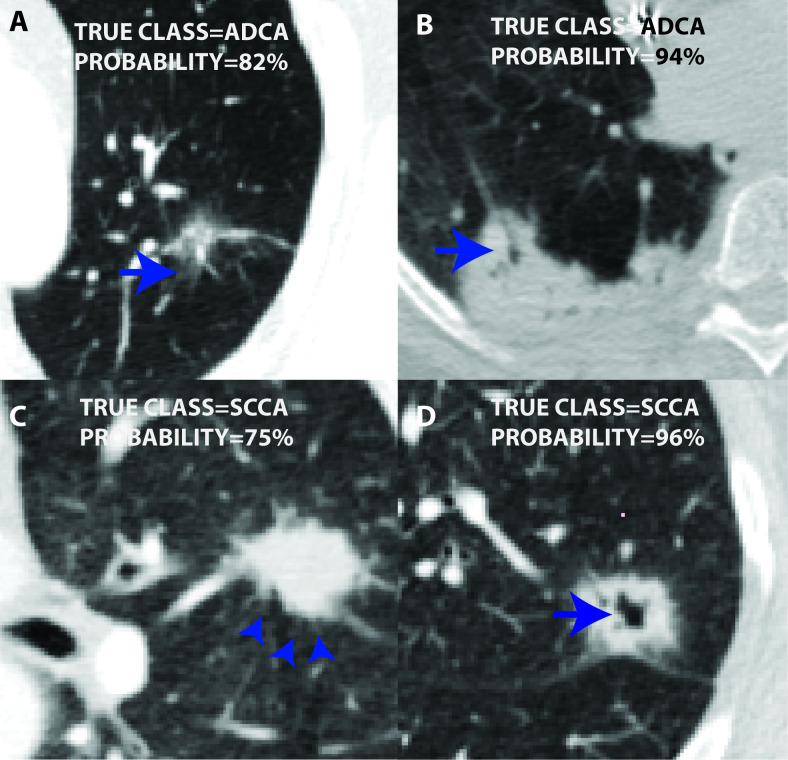
Figure showing two cases of ADCA (A, B), and two of SCCA (C, D). All cases were assigned high probability of respective histologies by the RF-sem model (inset). Among other semantic features, these tumours displayed features well known for ADCA, *i.e.* ground-glass component (arrow in A) and air bronchogram (arrow in B), and for SCCA, *i.e.* spiculation (arrow in C) and cavitation (arrow in D). Since spiculation was not strongly correlated with SCCA histopathology, the RF-sem model used absence of ADCA-specific features in C, although the overall confidence for SCCA (probability = 75%) was relatively lower. ADCA,adenocarcinoma; SCCA, squamous cell carcinoma.

The semantic random forest (RF-sem) performed equally well on training and test data sets with AUC of 0.78 and 0.82 respective (Figure 2). The radiomics-only and combined models gave perfect tumour subtype discrimination on the training data (AUC 1), but very low performance on validation data of AUC 0.5 and 0.56 respectively - similar to random chance (Figure 2). The OOB error (derived during model training) of RF-sem (25.5%) was also lower than that of RF-rad (40.6%) and RF-all (37.7%). Figure 3 shows example tumours of each type with class probabilities, highlighting the probabilistic nature of the random forest model that can be exploited in clinical decision-making to balance probability of tumour type against individual patient circumstances.

In terms variable importance, air bronchogram (MDA = 0.039), ground-glass component (MDA = 0.023), and cavitation (MDA = 0.019) were the top-ranking semantic variables, whereas tumour location, spiculation, and tumour margins did not have any discriminatory value. Of the radiomic variables, the highest ranking variables were grey-level size-zone matrix (GLSZM) short zone low intensity emphasis (GLSZM-SZLIE; MDA = 0.005), co-efficient of variation (MDA = 0.004), and neighbourhood grey-tone difference matrix (NGTDM) coarseness (MDA = 0.003). Variable importance of semantic features and top 10 ranking radiomic features (total = 756) is given in Table 4.

**Table 4.** Variable importance determined by random forests classifier using MDA

**Variable**	**MDA**
**Semantic features**	
Air bronchogram	0.039
Ground-glass component	0.023
Cavitation	0.019
Satellite nodules	0.015
Airway thickening	0.008
Pleural indentation	0.006
Emphysema	0.004
Pseudocavitation	0.002
Location	−0.002^a^
Spiculation	−0.005
Margin	−0.011
**Radiomic features**	
db1 LLL GLSZM Short Zone Low intensity emphasis	0.005
db1 HLH Coefficient of Variation	0.004
db1 LLL NGTDM Coarseness	0.003
db1 HHH GLCM Cluster Shade	0.003
db1 HHH NGTDM Coarseness	0.003
db1 HHH GLCM Correlation	0.003
NGTDM Contrast	0.003
Maximum intensity	0.003
db1 HHL Coefficient of Variation	0.002

GLCM, Grey-level cooccurence matrix; GLSZM, Grey-level size zone matrix; MDA, mean decrease in accuracy;NGTDM, Neighbourhood grey-tone difference matrix.

A high MDA score of a variable corresponds to greater predictive power.

a Negative MDA means the variable did not perform better than random chance. MDA = Mean decrease in accuracy. Note: Only the top 10 radiomic features are given here. For full table, please see supplemental file.

## Discussion

We developed three NSCLC classification models. RF-sem utilised semantic features obtained by consensus between two thoracic radiologists from training data and by a separate radiologist, from the validation data. RF-rad was based on computer-aided extraction of radiomic features from CT images of NSCLCs, whereas RF-all was a combination of semantic and radiomic features. RF-sem performed well on both training and validation data despite both data sets having been annotated by separate radiologists, indicating the robustness of random forests models developed with semantic features to inter-observer variability. RF-rad and RF-all gave perfect predictions on training data but performed no better than random guess on validation data—indicating a high degree of overfitting of random forests developed using radiomic features.

We found several semantic features highly predictive of NSCLC subtype (Table 3), of which air-bronchogram, ground-glass component, cavitation, and satellite nodules ranked highest in terms of discriminatory capability (Table 4). Our findings regarding the relative proportions of the various semantic features support previously reported trends, with a few differences^13,30–32^: Several clinical variables including older age, male gender, and smoking history are known to be more frequent in SCCA, in addition to semantic features such as spiculation and central location.^32^ In our cohort, none of these variables were significantly different between ADCA and SCCA and did not make a substantial contribution to the classifier.

The most important radiomic features in our study were GLSZM-SZLIE (MDA = 0.005), coefficient of variation (MDA = 0.004), and NGTDM coarseness (MDA = 0.003). The biologic counterparts of these features are poorly understood; here we attempt an intuitive explanation of what these features might represent in tumour CT images: The GLSZM, described originally for texture characterisation of cell nuclei,^33^ quantifies image heterogeneity in terms of zones of contiguous voxels sharing the same grey level intensity. A relatively homogeneous tumour would have large zones of voxels sharing similar grey level intensity and vice versa. The derived quantity GLSZM-SZLIE, as the name implies, would be expected to be high in tumours with heterogeneous distribution of low grey-level (*e.g.* ground-glass density) voxels. NGTDM coarseness, originally tested on various natural (*e.g.* pebbles, grass) and synthetic materials (*e.g.* cloth),^34^ would be high in tumours exhibiting similar intensities in neighbouring voxels with a low spatial rate of change in voxel intensities. In other words, they would comprise clusters of similar-intensity voxels which would stand out against the background and give a ‘coarse’ appearing texture to the tumour. Coefficient of variation (ratio of standard deviation over mean) is a first-order statistical texture feature which is high in tumours exhibiting high variation in grey-level intensities and low mean intensities. All three features were slightly more common in ADCAs *vs* SCCAs in our cohort.

A few authors have previously explored radiomics in NSCLC classification: In their proof of concept study, Basu et al trained a classifier (accuracy: 68%) on CT-derived radiomic features from 74 cases of NSCLC.^7^ Their study focused on differentiating the efficacy of 2D radiomic features versus 3D radiomic features and presented a comparison of various model categories including random forests, support vector machines, decision trees, and nearest neighbours. Their best model accuracy of 68% was obtained by employing all 215 features in a leave-one-out cross-validation scheme. However, the authors did not report the best performing variables and a comparison with our radiomic features can therefore not be performed. Two recent studies done by Wu et al. (*n* = 300) and Zhu et al (*n* = 129) have reported higher performance of radiomics-models (AUC 0.72 and 0.9 respectively).^6,8^ Other than that neither study compared radiomic features with semantic features, the most important difference between our study and either two is that the subset of highest performing radiomic features is different in all three studies. It is possible that since there are hundreds of radiomic features with majority inter-correlated, some of the different high-ranking features might merely be variations of the same feature. A second possibility is that some of the radiomic models developed by other authors may have overfit, as seen in our study, although Wu et al used an external validation cohort making this unlikely in their study. Overfitting is a common design problem in ML studies, especially in studies with a large number of variables with respect to cases and lack of external validation cohort. Radiomics is doubly challenged in gaining widespread acceptance due to the common use of hundreds of variables and issues surrounding reproducibility, although efforts are underway to standardise radiomics.^35^

Our study has several potential limitations: Because this was a CT study, we could not completely eliminate the possibility of including small regions of normal tissue, *e.g.* opacification due to adjacent atelectasis. However, we minimised such cases by excluding lesions that were difficult to delineate from adjacent collapsed lung. As a result, there may have been an under representation of centrally located SCCAs because such tumours were frequently inseparable from adjacent atelectasis. Central location is a known feature of SCCAs and including more centrally located tumours, expected to be majority SCCA, may have improved model performance.^33^ Secondly, as in most radiomics studies, our original radiomic feature space comprised a large number (*n* = 756) of features derived from CT scans with varying data acquisition parameters, especially those obtained from TCIA. Radiomic features are variable in terms of reproducibility and are dependent on tumour segmentation and image post-processing steps.^27^ Hence, we believe that future studies using a more refined selection of radiomic features, especially features engineered specifically for chosen classification tasks, may provide more useful results.

## Conclusions

Our study showed that non-invasive classification of NSCLCs using semantic features is possible and can be done with good accuracy (AUC: 0.82) using machine learning algorithms. However, CT-scan radiomic features performed poorly on independent validation data (AUC 0.5 and 0.56 for RF-tex and RF-all respectively), despite perfect classification on test data, and may be unsuitable for this task.
